# Digging Deeper to Diagnose: Cardiac Tamponade Following Tunneled Dialysis Catheter Placement

**DOI:** 10.7759/cureus.70317

**Published:** 2024-09-27

**Authors:** Esther H Kim, Priyanka Moolchandani, Satya Patel

**Affiliations:** 1 Internal Medicine, University of California Los Angeles, Los Angeles, USA

**Keywords:** cardiac tamponade, cardiac tamponade, dialysis, hemopericardium, pericardial effusion, tunneled dialysis catheter

## Abstract

Central venous catheter (CVC) placement is a common medical intervention in hospitalized patients associated with a host of complications, including cardiac tamponade. Here, we describe a case of a 61-year-old male with end-stage renal disease on hemodialysis via a right internal jugular tunneled dialysis catheter who presented to the emergency room for hypoxia at his skilled nursing facility. He had been discharged three days prior for treatment of a Methicillin-resistant Staphylococcus aureus (MRSA) neck abscess, during which an uncomplicated right internal jugular tunneled dialysis catheter exchange was performed one day prior to discharge. On admission, bedside point-of-care ultrasound (POCUS) showed a pericardial effusion without tamponade physiology. While receiving hemodialysis on his second day of admission, he was noted to have new hypotension, and repeat POCUS was concerning for tamponade. An urgent pericardiocentesis was performed with 895 mLs of serosanguinous drainage, followed by an additional 1400 mLs of serosanguinous drainage over the next 48 hours. Interventional radiology noted a contrast leak at the distal superior vena cava at the cavoatrial junction and suspected that the etiology for hemopericardium was an endovascular injury from tunneled dialysis catheter placement.

## Introduction

Cardiac tamponade is a medical emergency that results from the accumulation of pericardial fluid causing reduced diastolic filling and potential for decompensation into cardiogenic shock [1]. Iatrogenic causes either from medical or procedural intervention account for over 20% of tamponade cases, and of these, central venous catheter (CVC) placement-related tamponade events are extremely rare, with an incidence ranging from 0.0001% to 1.4%. These are often fatal, with a mortality rate of 65-100% [2,3]. Since CVC placement is one of the most common invasive procedures performed with over five million CVCs placed each year in the United States [4], the complications are important for internists to know. We present a case of cardiac tamponade presenting five days after an uncomplicated tunneled dialysis catheter exchange.

## Case presentation

A 61-year-old male with a history of end-stage renal disease on hemodialysis via a right internal jugular tunneled dialysis catheter was admitted for an oxygen saturation of 85% on room air at his skilled nursing facility. He had been discharged three days prior for treatment of a methicillin-resistant *Staphylococcus aureus* (MRSA) neck abscess that did not involve his tunneled dialysis catheter site or adjacent vascular structures.

During the prior hospitalization, it was noted that blood flow during hemodialysis sessions was sluggish; therefore, a tunneled dialysis catheter exchange over a wire was completed one day prior to discharge. During the catheter exchange, a superior vena cavogram showed a superior vena cava fibrin sheath that was disrupted via balloon angioplasty, after which a new dialysis catheter was inserted in the wire. His last dialysis session was three days before admission per his regular schedule, and his documented respiratory status on discharge was unlabored, with an oxygen saturation of 98% on room air.

On admission, vitals were notable for a heart rate of 98 beats, blood pressure of 85/60, and respiratory rate of 30 breaths per minute. He was briefly on a high-flow nasal cannula for tachypnea without desaturations and was quickly weaned to a 1L nasal cannula without intervention. The physical exam was unremarkable, aside from mild tachypnea. Admission laboratory studies are described in Table 1.

**Table 1:** Lab findings on admission WBC: White Blood Cell Count, Hgb: Hemoglobin, BUN: Blood Urea Nitrogen

Laboratory Study	Result	Normal Range
WBC	11,000 per uL	4,500 to 11,000 per uL
Hgb	7.5 g/dL	13.5 to 14.5 g/dL
BUN	68 mg/dL	0-25 mg/dL
Troponin	<0.01 ng/mL	0-0.04 ng/mL
Lactate	2 mmol/L	0.5-2.2 mmol/L

**Figure 1:**
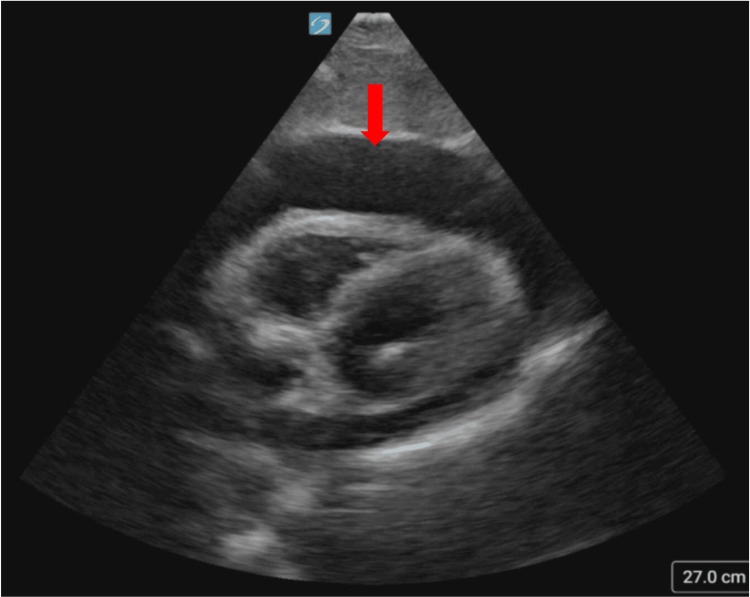
Point-of-care ultrasound on admission, with the arrow indicating the large pericardial effusion

As part of a comprehensive workup in the emergency room, computed tomography of the abdomen and pelvis was performed, showing a moderate-to-large pericardial effusion, and bedside point-of-care ultrasound (POCUS) showed a large pericardial effusion without right atrial or ventricular collapse during diastole, which would be suggestive of cardiac tamponade (Figure 1). The patient was given 500 mL of lactated Ringers solution intravenously, with an improvement in blood pressure to 115/75.

Due to the potential contribution of the effusion from uremic pericarditis despite effective hemodialysis, the frequency of the patient's hemodialysis was intensified. The following morning, the patient was receiving hemodialysis when he was noted to have a systolic blood pressure in the 70s. Electrocardiogram demonstrated sinus tachycardia without PR segment depression or electrical alternans. POCUS performed at that time demonstrated right ventricular diastolic collapse, concerning for cardiac tamponade. Cardiology was consulted, and an urgent pericardiocentesis under fluoroscopic and echocardiographic guidance yielded 895 mL of serosanguinous fluid, and a drain was left in place. On postoperative day 1, there was an additional 650 mL of serosanguinous output. Hemoglobin dropped to 6.9 g/dL and further decreased to 6.5 g/dL despite the administration of one unit of packed red blood cells (pRBCs). An additional unit of blood was given with an appropriate increase in hemoglobin. On postoperative day 3, the patient was noted again with hypotension and low drain output. The pericardial drain was exchanged, and blood clots were noted in the drain with 750 mL serosanguinous fluid output. Blood pressure improved from 72/26 to 130/54 after the exchange. Due to ongoing blood loss anemia, three additional units of pRBCs were transfused. Although his hemodialysis was thought to be effective, there was a concern for hemorrhagic effusion in the context of uremia, therefore the patient continued undergoing hemodialysis through his tunneled dialysis catheter. The persistent pericardial drain output and anemia prompted a transfer to an outside hospital for consideration of a pericardial window with cardiac surgery adjuncts, including potential ECMO and bypass.

At the outside hospital, interventional radiology was consulted for a superior vena cava venogram due to concern for complications related to the tunneled dialysis catheter. It noted a contrast leak at the distal superior vena cava at the cavo-atrial junction. Interventional radiology was unable to stent due to the risk of complications from stenting at the level of the right atrium.

Cardiothoracic surgery was consulted, but a repeat transthoracic echocardiogram showed resolution of pericardial effusion, therefore no further intervention was pursued. The patient's transfusion requirements declined, and he was subsequently discharged home safely, continuing hemodialysis through his tunneled dialysis catheter.

## Discussion

Central venous catheter (CVC)-associated cardiac tamponade is rare with a less than 1% incidence [2,3], though this assessment may be an underestimate due to the high mortality of this complication. Notably, the literature does not distinguish the rates of cardiac tamponade from tunneled versus non-tunneled CVCs. CVC-associated tamponade can occur in one of two ways. First, direct puncture of the cardiac or vessel wall during the procedure can cause tamponade within minutes to hours. Alternatively, catheter erosion through the vessel wall over time can cause tissue necrosis and subsequent perforation, most commonly at the superior vena cava (SVC) or the right atrium (RA) or ventricle [5]. The latter process can manifest more insidiously over days to several months [6].

There are several known risk factors for CVC-associated tamponade. Peripherally inserted central catheters have been found to carry greater risk than centrally inserted catheters for cardiac tamponade. This is in part thought to be due to the movement of the catheter tip with positional changes of the arms, neck, and head causing endothelial irritation [7]. The administration of hyperosmolar infusions, such as total parenteral nutrition, has been shown to increase the erosion of vessel walls and thus increase the risk of tamponade [7]. Prior CVC placement and CVC exchanges has been shown to increase the risk of tamponade [8]. Radiographic identification of catheter positioning should always be reviewed, with special attention to the catheter tip, which should be positioned 2-3 cm above the SVC-RA junction since the visceral pericardium extends above the SVC up to 3 cm [9]. Still, confirmation of position does not guarantee avoidance of vascular complications of CVC placement [10].

CVC-associated tamponade should be considered as part of the illness script for new or enlarging pericardial effusion in patients with central venous catheters. Presenting symptoms are often non-specific, including nausea, dyspnea, chest pain, hypotension, and tachycardia. Our patient presented with a chief complaint of hypoxia at his nursing facility, but on admission, he was notably mildly tachycardic, tachypneic, and hypotensive compared to baseline without true hypoxia. Hemodialysis was pursued due to concern for uremic pericarditis in the setting of his renal disease, though his blood urea nitrogen (BUN) was stable at baseline. For rapidly accumulating pericardial effusion, iatrogenesis from a vascular intervention should be considered. Once the diagnosis of a pericardial effusion is made, it is critical to determine if the effusion is leading to cardiac tamponade, which can be assessed in various ways. ECG findings that are specific to cardiac tamponade include PR segment depressions, low-voltage QRS complexes, and electrical alternans [11,12]. Pulsus paradoxus, a decline in the systolic blood pressure >10 mmHg with inspiration, is relatively specific but can be difficult to ascertain for a variety of reasons, including a lack of comfort with conducting the exam technique appropriately and sometimes a lack of manual sphygmomanometers in the care setting [11,12].

Management of CVC-associated tamponade first includes discontinuing any infusion through the catheter or, in our case, discontinuation of hemodialysis, which contributed to tamponade from reduced preload [2]. In our case, the hemodialysis was initially intensified due to concern for potential concern for uremic pericarditis despite consistent hemodialysis, which only exacerbated his condition. If symptoms do not resolve, the next step would be the removal of the central venous catheter and pericardiocentesis, followed by thoracotomy if unsuccessful [7-13]. In our case, a pericardial drain was left in place, which subsequently developed blood clots and caused repeat cardiac tamponade on postoperative day 3. Another case report of CVC-associated tamponade was initially managed with a pericardial drain, then as with our patient, recurrent tamponade occurred, necessitating an emergent pericardial window [14].

## Conclusions

Cardiac tamponade from central venous catheter placement is a rare but well-documented and fatal complication. We presented a case of hemopericardium causing cardiac tamponade, which was ultimately suspected to be caused by endovascular injury from tunneled dialysis catheter placement five days prior to the tamponade event. The tempo of this illness can be acute within minutes to chronic, even presenting months later. Clinicians should consider iatrogenic injury, and specifically hemopericardium, as a potential etiology to explain new pericardial effusions in patients with tunneled dialysis catheters in place. 
